# AI in Scholarly Communication

**Published:** 2024-03

**Authors:** Zrinka Pongrac Habdija

**Affiliations:** Linguistic Editor at Food Technology and Biotechnology; University of Zagreb, Faculty of Food Technology and Biotechnology, Pierottijeva 6, Zagreb, Croatia

The modern concept of artificial intelligence (AI) has been with us for decades, but with the recent launch of ChatGPT, it has rapidly captured our minds and found its way into almost every aspect of our lives. From Alan Turing to large language models (LLMs) like ChatGPT, we have come a long way that it is already hard to imagine our lives without the use of AI and it will certainly affect jobs in all walks of life.

In scholarly communication, there are many steps in the process from the formation of an idea for a study to the completion of a scientific paper and almost every step of the way can be aided by an AI tool. Today, there are even apps that can help you find a topic on which to focus your study. So if you run out of ideas, there is an AI help to your rescue. Apart from that, authors can use numerous language tools to help them express their thoughts more clearly, formatting tools to prepare papers, reviewers can use tools to help them summarise their reviews, and editors use tools to select reviewers, check submissions for originality and text overlapping, and much more. As opportunities for AI tools in publishing increase (and all parties involved are tempted to use them to at least help speed up routine tasks), they also bring challenges and a number of questions of integrity, ethics and rights that need to be addressed. Legislation often fails to keep up with the latest inventions and technologies, so is the case here. Laws regulating the use of AI are scarce; by the end of 2023, the European Union issued EU AI Act, the first regulation on artificial intelligence, which has been adopted as I am writing this text, *i.e.* on 13 March 2024 (*1*). This legislation attempts to manage the risks of AI. One of its main goals is to prevent harmful outcomes of its use to people and environment. It aims to protect fundamental rights, democracy, the rule of law and environmental sustainability from high-risk AI, while promoting innovation and establishing Europe as a leader in this field (*1*). China already has a patchwork of laws to control the use of AI for commercial purposes, while in the USA the preparation of legislation is in progress (*2*).

The EU Act is comprehensive and it does not bring specific recommendations for use of AI tools in scientific publishing. With the lack of legislation that would give recommendations on do’s and don’ts, publishers have come up with their own sets of recommendations and guidelines. Fast growth of generative AI applications has resulted in a number of concerns, among the main being authorship issues as well as legal copyright and research integrity issues. As of October 2023, of the 100 largest publishers and 100 highly ranked journals, 24 % of publishers and 87 % of journals had released guidelines on how generative AI could be used (*3*), with slightly different views among them on how the tools could be applied, but 96 and 98 %, respectively, prohibited the inclusion of generative AI as an author. American Association for the Advancement of Science (publisher of prestigious Science family of journals) was among first to include into their editorial policies a statement on the authorship and AI tools and their use in the preparation of text and figures. According to their statement (*4*), AI-assisted technologies, such as LLMs, chatbots and image creators do not meet the criteria for authorship and may not be listed as authors and co-authors, nor can the sources cited be (co)authored by AI tools. Also, AI-generated images are not permitted without permission from the editors, who may grant exceptions in certain situations which are evaluated on a case-by-case basis.

Springer Nature also monitor closely the developments in this area and are ready to adjust their policies when appropriate. Their policies refer to: (*i*) AI authorship, (*ii*) generative AI images and (*iii*) AI use by peer reviewers (*5*). Briefly, ad (*i*) LLMs do not satisfy authorship criteria, and their use should be clearly disclosed in the methods section or a suitable alternative paragraph of a manuscript. Ad (*ii*), the use of AI-generated images is not permitted; however, non-generative machine learning tools that are used to manipulate, combine or enhance existing images need to be disclosed appropriately upon submission. Ad (*iii*), a role of peer reviewers is vital in scientific publishing as their expert evaluations ensure publication of valid and credible research. They carry great responsibility and are accountable for the accuracy of their reports. That is why any use of tools needs to be transparent, as these tools have many limitations and need to be used with utmost care.

In Europe, Elsevier has issued a policy on the use of generative AI and AI-assisted technologies, which aims to provide transparency and guidance to authors, reviewers, editors and readers (*6*). The gist is similar, AI-assisted tools may be used, transparently, to improve readability and language of the work, but they cannot be listed as authors. They cannot replace humans in making decisions, conclusions or clinical recommendations.

COPE (Committee on Publication Ethics), a leading umbrella organisation, offers support to publishers, editors, readers, researchers and their institutions, mainly through education, resources and support on matters of ethics and good publication practices. COPE also states that AI tools cannot be listed as an author of a paper (*7*).

Clearly, among ethical issues, the matter of authorship is one of the primary concerns of scientific journals. Authorship is important as it implies responsibility and accountability for published work. It also has important academic, social and even financial implications. That is why many journals publish information about the contribution of each person named as an author of a study. Editors are encouraged to implement a contributorship policy that will help clarify the role of each contributor and at least partly remove ambiguity regarding the amount and quality of contribution. The International Committee of Medical Journal Editors (ICMJE) has thus developed criteria for authorship that distinguish authors from other contributors. According to ICMJE recommendations (*8*), authorship is based on the following 4 criteria:

- Substantial contributions to the conception or design of the work; or the acquisition, analysis, or interpretation of data for the work; AND
- Drafting the work or reviewing it critically for important intellectual content; AND
- Final approval of the version to be published; AND
- Agreement to be accountable for all aspects of the work in ensuring that questions related to the accuracy or integrity of any part of the work are appropriately investigated and resolved.

More importantly, besides being accountable for the parts of the work done, an author should also be able to identify which co-authors are responsible for specific other parts of the work. In addition, authors should have confidence in the integrity of the contributions of their co-authors. All those designated as authors should meet all four criteria for authorship, and all who meet the four criteria should be identified as authors. Based on these recommendations by ICMJE, AI tools and AI-assisted LLM tools do not meet the criteria for authorship and cannot therefore be considered authors (*8*). Similar, but according to some more contemporary, are the 14 contributor roles laid out by Contributor Roles Taxonomy (CRediT) (*9*). The roles given in the taxonomy are not limited to traditional authorship roles, but intend to cover all the work that enables the production of scholarly publications. The contributions are machine readable and can be incorporated in article XML files. Not surprisingly, the number of highly ranked journals implementing CRediT are continually increasing.

*Food Technology and Biotechnology* journal follows the recommendations of COPE and ICMJE and our policy (*10*) concerning the authorship criteria does not allow listing an AI tool as an author of submissions to our journal. A recent survey conducted among 68 editorial offices in Croatia showed that 66 % of journals still do not have any recommendations about the use of AI tools in their instructions to authors, but that 29 % of them are considering putting up some guidelines (*11*).

Another great concern with the use of generative AI and AI-assisted tools is in figures and images. Most journals agree that the use of such tools to manipulate images is not permitted. Manipulations that would alter the interpretation of an image, including removing of adding a feature are strictly prohibited. Only adjustments of brightness, contrast and colour balance are allowed, as long as they do not obscure or eliminate any original information.

Other uses of LLMs or AI-assisted tools are generally not forbidden, but the emphasis is on clear and transparent disclosure. Recommendations vary slightly, but most publishers agree that, for example, language tools like spelling and grammar checkers (*e.g.* Grammarly, InstaText and PerfectIt) that help non-native speakers express their thoughts better in English and improve readability are permitted even without disclosure. The last one even boasts that it is not AI-based tool (*12*), so your sensitive information is safe. Another example of permitted tools are reference managers such as Zenodo, Mendeley, EndNote and others. They are very useful in collecting and organising references, and they can be used without disclosure.

Opinions on the benefits of use of LLM tools vary. While some believe that the greatest benefit of generative AI was help researchers whose first language is not English and thus improve equity in science by reducing the number of rejected papers due to language issues, others fear that easier access to generative AI tools will compromise research integrity as it is easy to use them produce a large amount of poor quality texts in a very short time (*13*).

General recommendation to authors and editors is to use the currently permitted tools responsibly. Editorial decisions about rejection or acceptance of submissions should not be brought based only on an AI recommendation. Only editors must take responsibility for editorial decisions. Authors when using different tools, must check the outputs they obtain, as many generative AI tools can produce convincing texts that on the surface seem credible but contain false information, which is not only unethical but can even be dangerous. AI is known to lie well. Another concern is that they can exacerbate the problem of the so-called paper mills, the sole purpose of which is to boost publishing output and increase profit.

It is not uncommon in human history that new discoveries, new gadgets, new tools bring with them mixed feelings, both of excitement and concern. Whenever people develop an invention intended to improve our lives, someone immediately comes up with ways to abuse it. A bit like superheroes and villains, each superhero has his or her nemesis. Since technologies are not going away, people will keep on inventing and developing stuff, we must learn to embrace them and not fear them. For that we need strong ethical regulations and mind set to fully enjoy what they can offer. We must work together on building firm foundations so that future generations can continue building on them and I believe, as someone recently wrote somewhere, that AI will not replace humans, it will only replace those who will not know how to use it.
